# Characterization and Pathogenicity of Novel Reassortment H6N6 Avian Influenza Viruses in Southern China

**DOI:** 10.1155/2024/4005909

**Published:** 2024-07-29

**Authors:** Puduo Zhu, Xudong He, Yiquan Chen, Zhuanqiang Yan, Qunhui Li, Qi Zhou, Wencheng Lin, Feng Chen

**Affiliations:** ^1^ College of Animal Science South China Agricultural University, Guangzhou 510642, China; ^2^ Wen's Food Group, Yunfu 527300, China

## Abstract

The H6N6 avian influenza virus (AIV) subtype is one of the most frequently isolated subtypes in poultry, and it has a broad host range. Some strains can overcome species barriers for transmission and infect humans. Increased affinity for human-type receptors is a key factor in this process. In this study, two H6N6 AIV strains originating from five different clades, in which amino acid 226 of hemagglutinin was mutated from glutamine to lysine, were isolated from ducks. The receptor-binding preference and pathogenicity of the two strains in poultry and mice were evaluated. The results indicated that the DK/GD/W3 strain can bind to both *α*-2,6 and *α*-2,3 receptors, whereas the DK/GD/L31 strain maintained affinity toward avian-origin *α*-2,3 receptors, highlighting differences in receptor tropism and pathogenicity to different hosts for two H6N6 strains with the same genetic background. These findings have revealed the complex recombination characteristics and molecular characteristics of H6N6 circulating strains in the environment and underscored the importance of continuous surveillance of this subtype for livestock and poultry health as well as human safety.

## 1. Introduction

The H6N6 avian influenza virus (AIV) is a low-pathogenic virus belonging to the *Orthomyxoviridae* family. Each genome consists of eight negative-sense single-stranded RNA segments, and the antigenic differences in surface proteins (hemagglutinin (HA) and neuraminidase (NA)) serve as the basis for genotype classification [1]. The H6N6 virus can infect various birds, including poultry, migratory birds, and waterfowl, as well as pigs [2]. It typically causes mild or asymptomatic symptoms in the host. However, extensive transmission among several bird hosts and interactions with different hosts can lead to genetic recombination and shift in H6 subtype viruses, resulting in increased pathogenicity. Based on the genetic characteristics of the HA gene, H6 AIVs are classified into the North American and Eurasian lineages [3]. The prevalence of H6 AIV in China is complex, and the virus is primarily categorized into five lineages, including the early lineage, W312-like, Group I, Group II, and Group III. Group I, represented by A/duck/Shantou/339/2000(H6N2), is mainly distributed among poultry in Hong Kong and Shantou. Group II, represented by A/wild duck/Shantou/2853/2003(H6N2), is found in coastal provinces such as Guangdong and Fujian. Group III, represented by A/duck/Hunan/573/2002(H6N2), includes H6 AIV and migratory bird strains in Hunan and Guangxi [4, 5, 6, 7].

Since 2005, the H6N6 subtype has been circulating in regions such as southwestern, central, and eastern China. Following the peak of its prevalence in 2015, the H6N6 subtype became predominant in southern China [8]. In 2011, a swine-origin H6N6 strain was isolated in Southern China [9]. The occurrence of the H6 subtype in different species was demonstrated an H6N1 infection was reported in humans in Taiwan in 2013. In addition, strains with the cleavage site PQIATR ^*∗*^G have been isolated from dogs [10, 11]. In 2014, the first case of human infection by H6N6 virus, which was accompanied by fever symptoms, was reported in mainland China. These events collectively emphasize the significant importance of continuous monitoring of H6N6 AIV for human health and public health safety.

In this study, we analyzed the genetic evolution and amino acid loci of the whole genomic sequence of two H6N6 AIVs isolated from ducks. We performed goose red blood cell (GRBC) agglutination and solid binding experiments according to the characteristics of HA amino acid loci and further explored the pathogenic effects of these two strains in different hosts.

## 2. Materials and Methods

### 2.1. Virus Isolation

A total of 529 swab samples were collected from September to December 2023, of which 150 were from Guangdong live poultry markets and 379 were from Muscovy duck farms. Cloacal swabs from ducks were collected in phosphate-buffered saline (PBS) containing dual antibiotics (2,000 units each of penicillin and streptomycin). After vortexing and centrifugation, the supernatant was filtered through a 0.22-*μ*m membrane and inoculated at 0.1-mL doses into 9–11-day-old specific pathogen-free (SPF) chicken embryos (provided by Guangdong Wen's DaHua Biotech Co., Ltd., Yunfu, China). After incubation at 37°C for 72 hr, the allantoic fluid was collected and stored at −80°C for further use.

### 2.2. Sequencing and Phylogenetic Analysis

Viral RNA was extracted from HA test-positive allantoic fluid using a BioTek DNA/RNA Extraction Kit. Specific primers for eight gene segments and the PrimeScript™ One Step RT-PCR Kit (Beijing Bioridge Biological Technology Co., Ltd., Beijing, China) were used to amplify the full-length gene segments by polymerase chain reaction (PCR). PCR products were cloned into the pMD19-T vector (Beijing Bioridge Biological Technology Co., Ltd.). Single colony were selected and sent to Shanghai Bioengineering Co., Ltd. (Shanghai, China) for sequencing. Nucleotide sequences were assembled using the SeqMan software within the DNASTAR Lasergene 11.0 package and submitted to GenBank under the accession numbers listed in Table 1. The obtained sequences were compared with the previously reported sequences of reference strains, all of which were sourced from the GASID and NCBI databases. Phylogenetic trees for the eight gene segments were constructed using the MEGA7.0 software using the neighbor-joining method with 1,000 bootstrap replicates. Then use the online test system NetNGlyc 1.0 (https://services.healthtech.dtu.dk/services/NetNGlyc-1.0/) to obtain the amino acid sequence of potential glycosylation sites which is analyzed.

### 2.3. HA Assay for Receptor Analysis

To test the receptor-binding specificity of the two viruses, we incubated 100 *μ*L of 10% GRBCs with 1.25 units of *α*-2,3-sialidase (TaKaRa, Shiga, Japan) at 37°C for 1 hr. The treated GRBCs were washed twice and adjusted to a working concentration of 0.5% using PBS. The viral fluid (50 *μ*L) was serially diluted with 50 *μ*L of PBS and mixed with 50 *μ*L of 0.5% GRBCs in a 96-well microtiter reaction plate. After incubation at room temperature for 15 min, the HA titers were determined. The A (H1N1)pdm2009 virus A/California/04/2009 (CA/04) and poultry H5N1 isolate A/mallard/Huadong/S/2005 (HD/05) were used as controls.

### 2.4. Solid-Phase Direct Binding Assay

To further test the receptor-binding characteristics of the aforementioned viruses, we validated the receptor properties using a solid-phase direct binding assay. The receptor analogs Neu5Aca2-3Galb1-4GlcNAcb (3SLN)-PAA-biotin and Neu5Ac*α*2-6GalNAc*α*-sp3-PAA-biotin (GlycoNZ, Auckland, New Zealand) were washed three times with PBS and then added at the same rate to a streptavidin-coated 96-well plate. After overnight blocking with 5% BSA, the plate was washed three times with 0.05% PBST. The virus was added to each well at 128 HA units, and the AIV universal monoclonal antibody was diluted in PBS. Horseradish peroxidase-conjugated mouse immunoglobulin G secondary antibody and a substrate solution containing tetramethylbenzidine were used for binding. The reaction was stopped by the addition of 1 M H_2_SO_4_, and absorbance was measured at 450 nm. Triplicate measurements were taken for each sample.

### 2.5. Mouse Experiment

Thirty-three 6-week-old female BALB/c mice (Guangdong Medical Laboratory Animal Center, Foshan, China) were randomly divided into three groups with 11 mice each group. The dosage was calculated on the basis of body weight, and the mice were mildly anesthetized using Zoletil® 50 (tiletamine hydrochloride and zolazepam hydrochloride). Each mouse was intranasally inoculated with a dose of 10^6^ EID_50_/50 *μ*L after anesthesia, whereas control mice were inoculated with 50 *μ*L of PBS. Three mice from each group were euthanized at 3 and 5 days post inoculation (dpi), and the spleen, lungs, kidneys, brain, nasal turbinates, and trachea were collected. Virus titration was conducted in SPF chicken embryos, and organ virus titers were calculated using the Reed–Muench method. The remaining five mice were observed continuously for 14 days, and their weight and survival rates were recorded.

### 2.6. Chicken Experiment

Twenty-four 4-week-old SPF chickens (Guangdong Wen's DaHua Biotech Co., Ltd.) were randomly divided into three groups with eight chickens each group. Each chicken was intranasally inoculated with 10^6^ EID_50_/200 *μ*L. Throat and cloacal swab samples were collected from each group at 2, 4, and 6 dpi. On day 5, three chickens from each group were randomly euthanized, and tissues, including the heart, liver, spleen, lungs, kidneys, duodenum, and trachea, were collected. Both swabs and tissue samples were subjected to virus titration in SPF chicken embryos, and viral titers were calculated using the Reed–Muench method. The remaining chickens were continuously observed for 14 days.

### 2.7. Duck Experiment

Thirty 14-day-old ducklings (Wen's Food Group Ltd., Yunfu, China) were randomly divided into three groups with 10 ducks each group. To ensure normal replication of the virus in Muscovy ducks, antibody negative was confirmed prior to the test. Each duckling was intranasally inoculated with 10^6^ EID_50_/200 *μ*L. Throat and cloacal swab samples were randomly collected from ducks at 1, 3, 5, and 7 dpi. On days 3 and 5, three ducks from each group were randomly euthanized, and tissues, including the heart, liver, spleen, lungs, kidneys, duodenum, and trachea, were collected. Both swabs and tissue samples were subjected to virus titration in SPF chicken embryos, and viral titers were calculated using the Reed–Muench method. The remaining ducks were continuously observed for 14 days.

### 2.8. Histopathological Analysis

At 3 and 5 dpi, lung tissues from mice and lung/trachea tissues from ducks were collected, and on day 5, lung and trachea tissues were collected from chickens. The tissues were fixed in 10% formalin for 120 hr at room temperature, sectioned into 5-*μ*m-thick paraffin sections, and deparaffinized after hydration. Sections were stained with hematoxylin and eosin (H&E).

### 2.9. Statistical Analysis

All data were statistically analyzed using GraphPad Prism version 8.0 software (GraphPad, La Jolla, CA, USA).

### 2.10. Gene Sequence Accession Numbers

The nucleotide sequences of the two isolated H6N6 AIV strains obtained in this study have been deposited in NCBI, and the accession numbers are listed in Table 1.

## 3. Results

### 3.1. Phylogenetic Analysis

**Figure 1:**
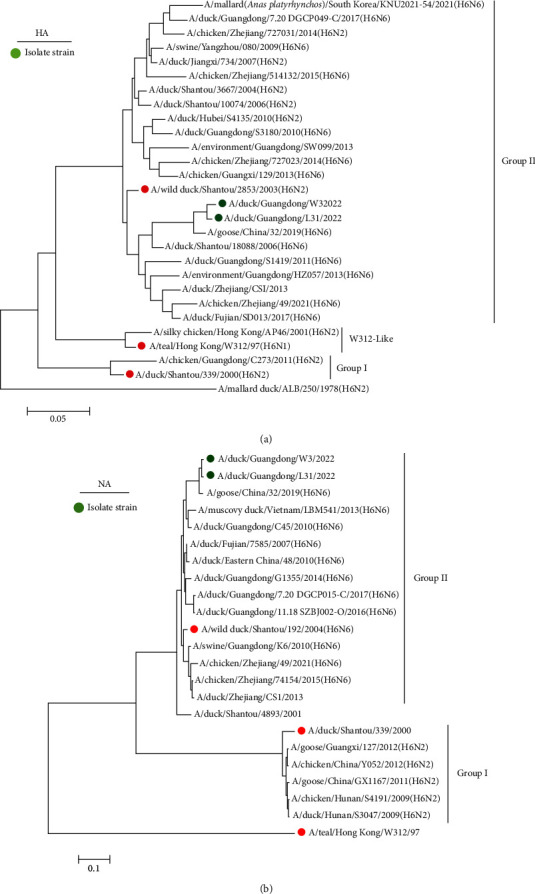
Phylogenetic analysis of hemagglutinin (HA) gene of H6 subtype influenza viruses (a) and phylogenetic tree of NA gene of N6 subtype influenza viruses (b). Isolate strain is shown in green solid circle.

In this study, the HA genes of the two viruses clustered within Group II alongside DK/GD/W3 and DK/GD/L31, respectively, and they grouped together with the goose-origin strain A/goose/China/32/2019(H6N6) with 97.35%–97.65% similarity (Table 1). The NA genes of both viruses also clustered within Group II, represented by A/wild duck/Shantou/192/2004(H6N6), exhibiting 90.1%–92.3% homology. This suggests that the two viruses share a similar NA gene origin (Figure 1).

**Figure 2:**
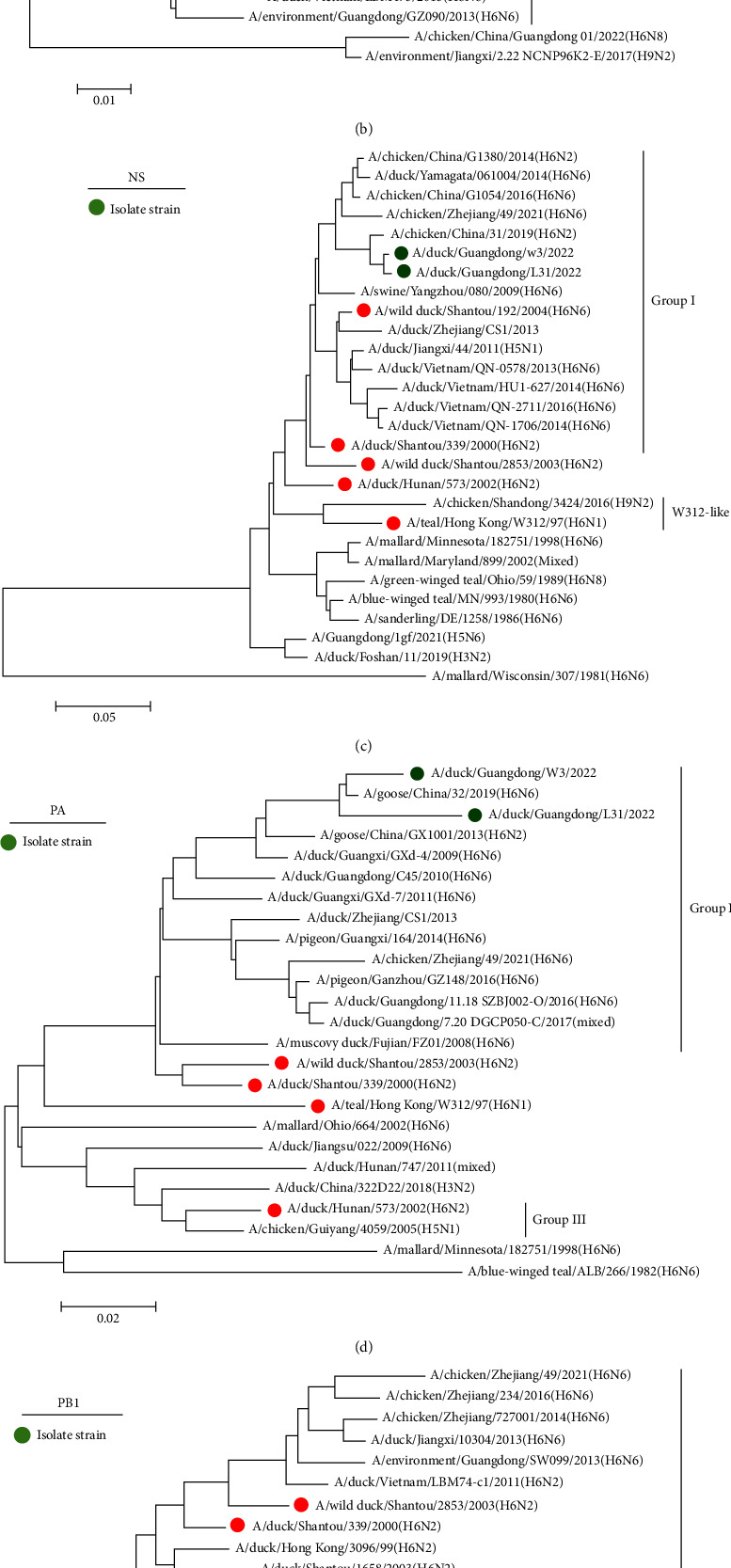
Phylogenetic analysis of M, NP, PA, NS, PB1, and PB2 gene. The M (a), NP (b), NS (c), PA (d), PB1 (e), and PB2 (f) genes of the H6 subtype AIVs from this study are indicated by green solid circle.

**Figure 3:**
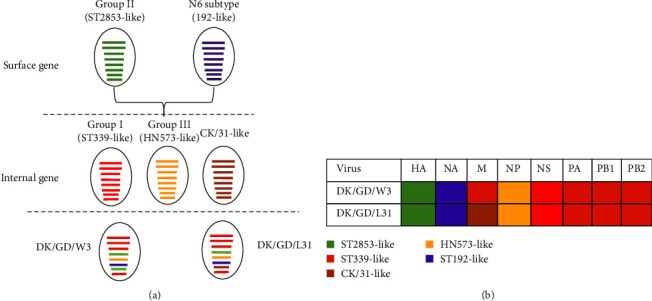
The genotypes of the four H6 subtype AIVs. The two H6 subtype AIVs were reassortment from the five subgroups, including ST2853-like, HN573-like, ST192-like, CK/31-Like, and ST339-like (a, b). And the rectangles of different sizes represent PB1, PB2, PA, HA, NP, NA, M, and NS genes from top to bottom (a, b).

Phylogenetic analysis of internal genes indicated that the PA and PB2 genes of the two viruses clustered within Group I, and these strains were closely related to H6N6 strains from Guangdong and Fujian. The PB1 and NS genes of both viruses also clustered in Group I, indicating a similar gene origin. The NP gene clustered in Group III. The M gene of the DK/GD/W3 strain clustered in Group I, whereas that of the DK/GD/L31 strain clustered with the chicken-origin H6N2 strain A/chicken/China/31/2019(H6N2), with high similarity (98.66%). These results suggest that the external genes HA and NA of the two viruses isolated in this study are closely related to waterfowl hosts. In this study, the surface gene source of the two isolates was the same and clustered together with the waterfowl H6N6 AIV strain. The six internal genes were highly similar to those of chicken and goose H6N6 strains. The gene source of the two isolates was more complex and showed different recombination characteristics (Figures 2 and 3).

### 3.2. Molecular Characteristics

Based on the sequence analysis results (Table 2), we found that the cleavage site of HA for both viruses is PQIETR↓G, and the sequence did not contain consecutive basic amino acids, consistent with the characteristics of low-pathogenicity AIVs [12, 13]. We detected mutations of 190Q and 226L (H3 numbering) in the receptor-binding site of HA in the DK/GD/W3 and DK/GD/L31 strains [14, 15]. Additionally, HA1 (26, 27, 39, 306, and 311) and HA2 (498 and 577) of both strains contained seven potential glycosylation sites, which were highly conserved.

Usually, the absence of the stalk region in NA indicates an enhancement of the virulence of viruses in mammals [17], but no amino acid deletions were found in the stalk region of NA of the two viruses isolated in this study. In addition, no mutations such as E119V and H274Y were detected, indicating that the two viruses are sensitive to NA inhibitors (Oseltamivir) [18, 19]. We detected seven potential glycosylation sites in NA from the DK/GD/W3 and DK/GD/L31 strains (40, 51, 67, 70, 146, 201, and 402).

Mutations such as E627K, D701N, and T271A in PB2 can enhance polymerase activity, thereby increasing the virulence and transmission of AIV in mammals [20, 21], which is considered a key factor in interspecies transmission [22, 23, 29]. However, no relevant mutations were found in the H6N6 viruses isolated in our study. The N31S mutation in M2 suggests viral resistance to M2 blockers and antiviral drugs such as amantadine. The I38M mutation in the PA protein can enhance the virulence of the virus in mice [27, 28], and mutations D92E and P42S in the NS protein can enhance the virus's ability to evade host antiviral cytokines [26]. In our study, both viruses carried the P42S mutation, indicating a trend of enhanced virulence in the host.

### 3.3. HA Assay

**Figure 4:**
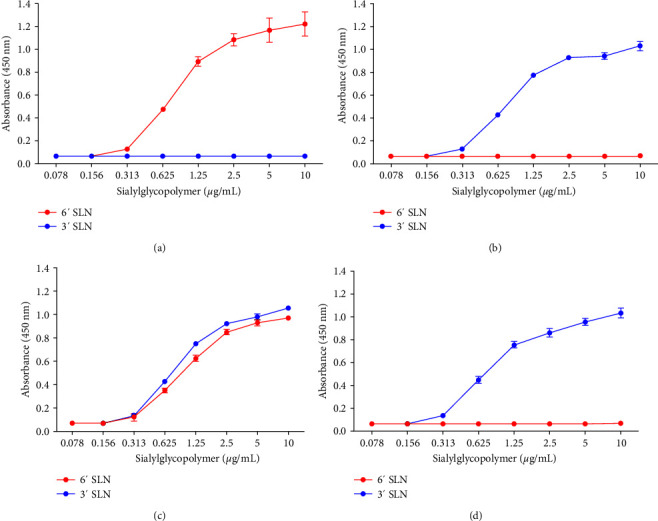
Solid-phase receptor-binding assay of human isolate A/California/04/2009 (a), poultry isolate A/mallard/Huadong/S/2005 (b), DK/GD/W3 (c), and DK/GD/L31 (d). Direct binding of viruses to sialylglycopolymers containing either 3′SLN-PAA or 6′SLN-PAA was measured. The color of the line indicates different synthetic sialylglycopolymers as follows: blue, 3′SLN-PAA, and red, 6′SLN-PAA, respectively.

In general, H6N6 AIVs exhibit low pathogenicity in poultry and limited ability to infect and spread in humans. One crucial factor restricting AIV infection in humans is the receptor specificity of HA. We treated GRBCs with a *α*-2,3–specific NA and virus solution. NA digestion should eliminate the HA activity of *α*-2,3-specific viruses, whereas viruses capable of binding to *α*-2,6 receptors should maintain HA activity in RBCs (Table 3). To further validate receptor specificity, a solid-phase direct binding assay was conducted. The results indicated that CA/04 and H5/Re-5 bind to *α*-2,6 and *α*-2,3 receptors, respectively. The DK/GD/L31 strain bound to *α*-2,3 receptors, whereas the DK/GD/W3 strain was capable of binding to both receptors simultaneously (Figure 4).

### 3.4. Mouse Experiment

**Figure 5:**
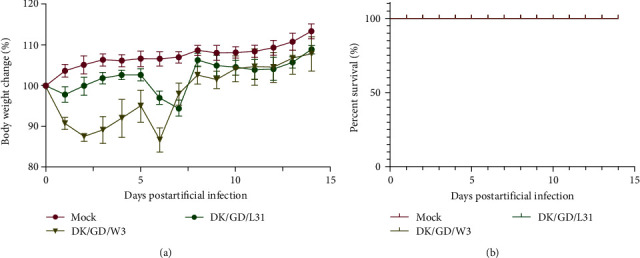
The pathogenicity of DK/GD/W3 and DK/GD/L31. BALB/c mice were challenged with 10^6^ EID50 virus. Body weight (a) and survival (b) were observed for 14 days.

To further explore the potential pathogenicity of the two viruses, mice were infected with a dose of 10^6^ EID_50_/50 *μ*L. The mice exhibited significant weight loss at 2–7 dpi, with mice infected with the DK/GD/W3 and DK/GD/L31 strains reaching their lowest weights at 6 and 7 dpi, respectively. Subsequently, the mice gradually regained their weight (Figure 5(a)). Overall, mice in the experimental group consistently had lower weights than those in the control group during the experiment. Compared to the findings in the blank control group, the weight loss rate in the DK/GD/W3-infected group exceeded that in the DK/GD/L31-infected group. No infected mice died in the experiment, indicating that the two viruses were not lethal to mice (Figure 5(b)).

To assess viral replication in mouse organs, we evaluated viral titers in the spleen, lungs, kidneys, brain, organs, nasal turbinate, and other tissues at 3 and 5 dpi. The results indicated that the virus efficiently replicated in the lungs, nasal turbinates, trachea, and other tissues. In addition, the DK/GD/W3 strain was detected in brain tissue. However, no virus was detected in the spleen or kidneys. In summary, both virus strains can infect mice and efficiently replicate in the upper respiratory tract. However, in terms of the weight loss rate and tissue viral load, the DK/GD/W3 strain exhibited greater pathogenicity than the DK/GD/L31 strain in mice (Table 4).

### 3.5. Chicken Experiment

To assess the pathogenicity of the H6N6 isolates in chickens, each group of eight chickens was intranasally inoculated with the viruses at a dose of 10^6^ EID_50_/100 *μ*L. In addition, three cohabiting chickens were introduced into each group at 1 dpi. Throat and cloacal swab samples were randomly collected from three inoculated chickens at 2, 4, and 6 dpi. At 5 dpi, the heart, liver, spleen, lung, kidney, duodenum, and trachea samples were collected from three chickens. The viral content in the swab samples and tissues was determined by chicken embryo inoculation. At 2 dpi, both strains were detected in oral and cloacal swabs from inoculated chickens. Only throat swabs from two inoculated chickens tested positive for the DK/GD/L31 strain at 4 dpi, and the DK/GD/W3 strain was not detected in throat samples. By 6 dpi, neither virus was detectable in swab samples (Table 5). To further investigate the pathogenicity of the viruses in SPF chickens, virus titration was performed on the collected organs. The results demonstrated that the viruses can replicate in the lungs and trachea. In addition, the DK/GD/W3 strain was detected in the kidneys. In summary, both duck-origin H6N6 subtype AIVs successfully infected chickens, but their pathogenicity was relatively weak, with lower viral content in swabs and tissues (Table 6).

### 3.6. Duck Experiment

We infected ducks with the same viral dose and method used in the chicken experiment. Swab samples were collected at 1, 3, 5, and 7 dpi, and organ tissues, including the heart, liver, spleen, lungs, kidneys, duodenum, and trachea, were collected at 3 and 5 dpi. SPF chicken embryos were inoculated to detect the viral load. The viruses were detected in throat and cloacal swabs, with the highest viral content observed at 3 and 5 dpi (Table 7). The viral content was relatively high in lung and tracheal tissue, and both viruses were was detected in the duodenum as well as the spleen in kidneys in some ducks (Table 8).

### 3.7. Tissue Pathological Analysis

**Figure 6:**
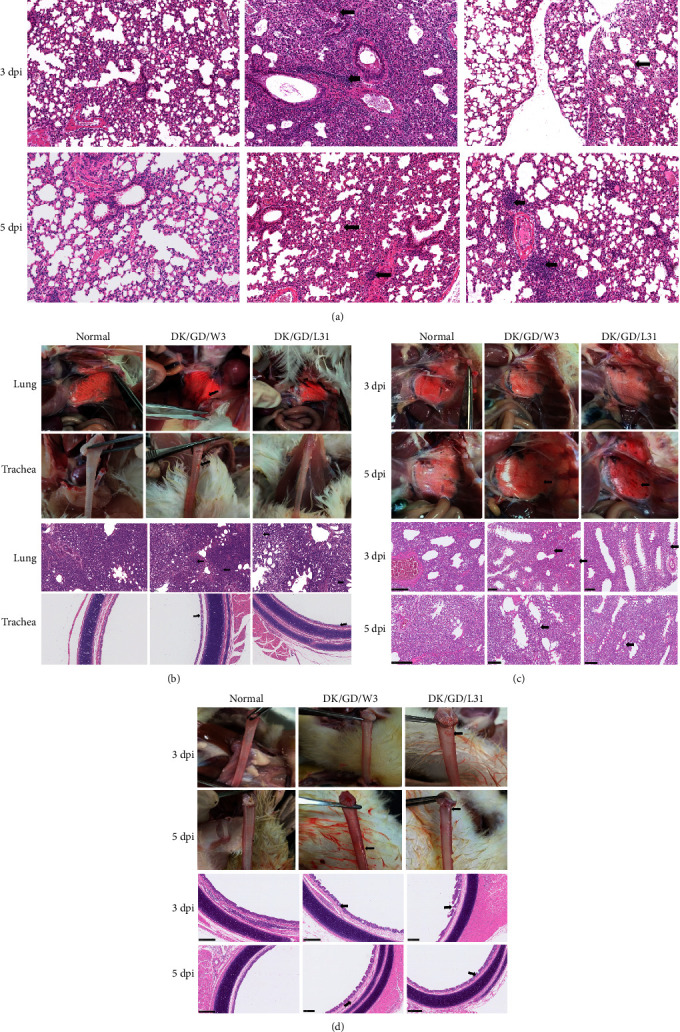
The pathogenicity of H6N6 isolate strain virus in mice was challenged with 10^6^ EID_50_ virus. Histopathological changes of hematoxylin and eosin (H&E)-stained tissue sections at 3 and 5 dpi of H6N6 isolate strain virus infected mice (a), at 5 dpi of H6N6 isolate strain virus infected chicken (b), and 3 and 5 dpi of H6N6 isolate strain virus infected duck (c, d). Magnification, ×200.

We prepared pathological sections to further observe the histopathological changes in the tissues of animals infected with the two viruses. The results revealed consolidation, widened alveoli, hemorrhage, congestion, and inflammatory cell infiltration in the lung tissues of mice infected for 3 or 5 days. In addition, a small amount of desquamation and thinning of bronchiolar epithelial cells were noted. These pathological changes indicate infection and varying degrees of damage in lung tissues, suggesting that the virus can replicate in mouse lung tissues and induce an inflammatory response (Figure 6(a)). At 5 dpi in chickens, partial areas of lung tissue displayed consolidation, widened alveolar septa, slight hemorrhage, expanded surrounding alveoli, and mild inflammatory cell infiltration. Chicken tracheal tissue exhibited increased proliferation of goblet cells, and submucosal inflammation was minimal. Histopathological changes in chicken tissues indicated minor damage to the trachea (Figure 6(b)). HE staining revealed mild alveolar expansion, widened septa, and a small amount of inflammatory cell infiltration at 3 and 5 dpi in the lung tissues of infected ducks. Duck tracheal tissue exhibited slight mucosal shedding and slightly loose connective tissue (Figures 6(c) and 6(d)).

## 4. Discussion

The transmission of AIVs in hosts relies on receptor characteristics, and expansion of the host range poses a risk as species barriers become compromised. In recent years, the discovery of numerous AIV subtypes in waterfowl, particularly the H6 subtype, has resulted in multiple genetic exchanges among H9N2, H7N9, and H5N1 strains, leading to significant outbreaks [30, 31]. In particular, recombination of internal genes is concerning, emphasizing the need for sustained monitoring of these viruses.

Over the past two decades, the H6N6 and H6N2 subtypes have become increasingly prevalent in southern China, and they have gradually become the predominant subtypes [32]. The observation of diverse origins of two viral gene segments in this study suggests that various factors, such as poultry farming practices, transportation, and centralized selling, as well as environmental conditions such as shared habitats and water sources for wild birds, contribute to the interaction and mutual transmission of AIVs in different regions. The results of this study highlighted the genetic characteristics, pathogenicity, and host specificity of two H6N6 AIVs isolated from ducks. Genetic analysis revealed that both viruses belonged to Group II in terms of HA and NA genes, displaying close relationships with other H6N6 strains identified in Guangdong and Fujian provinces. The internal genes, including PA, PB2, PB1, NP, and NS, were associated with H6N6 strains circulating in Guangdong and Fujian.

In general, the surface genes of the two strains were different from the internal genes. The DK/GD/W3 strain had four-element recombination characteristics, the DK/GD/L31 strain had five-element recombination characteristics, and the two strains showed genotype, highlighting the potential for recombination and cross-species transmission. Therefore, optimizing poultry farming conditions and implementing measures to minimize contact with wild birds are necessary to prevent and control the spread of these viruses.

Receptor-binding preference is crucial for the replication and transmission of influenza viruses. Human influenza viruses preferentially bind to *α*-2,6 receptors, whereas AIVs bind to *α*-2,3 receptors [33, 34]. Previous reports indicated that mutations in 226L and 228S lead to a preference for *α*-2,6 receptors in AIV; however, the factors that cause mutations at these sites and alter receptor-binding preferences remain unclear. Yan et al. [35] found that the E190Q/G, Q226L, and G228S mutations can shift virus host specificity, potentially increasing the ability of the viruses to bind to mouse and human lung epithelial cells and further enhancing their cross-species infectivity [3, 15, 16, 36].

In this study, we verified the pathogenicity of strains carrying a 226L mutation in mice, chickens, and ducks. The viruses replicated efficiently in the respiratory tracts of mice and ducks with evidence of viral shedding. The DK/GD/W3 strain in mouse lung and tracheal tissues exhibited virus titers as high as 4.9 ± 4.5 log_10_ EID_50_ and 3.98 ± 0.20 log_10_ EID_50_, respectively. The DK/GD/L31 strain showed virus titers of 4.1 ± 0.1 log_10_ EID_50_ and 3.81 ± 0.64 log_10_ EID_50_ in the same tissues. Viral shedding was detected in the duck reproductive tract and throat, reaching titers of 3.4 ± 0.72 log_10_ EID_50_ and 3.25 ± 0.15 log_10_ EID_50_ on the third and fifth days, respectively. However, virus titers were low in lung and tracheal tissues. In chicken swabs and tissue samples, the viral content was relatively low. In various pathogenicity experiments, the DK/GD/W3 strain exhibited higher pathogenicity than the DK/GD/L31 strain. Both viruses demonstrated the ability to breach the blood–brain barrier in mice and colonize the brain. In addition, they were detected in the kidneys of chickens and duodenum of ducks, exhibiting an organ tropism distinct from that of DK/GD/L31 strains. However, the viruses did not cause significant organ damage in the infected animals. The test results showed that the strain with the 226L mutation had the ability to infect mice, Muscovy ducks, and SPF chickens, and the detoxification within 1 week showed that the virus had a stronger ability to infect Muscovy ducks than SPF chickens. The virus content detection results showed that the virus could effectively replicate in the lungs, trachea, and turbinate bone of mice. The results indicated that the duck-derived H6N6 strain with 226L mutation had different infecting ability to different hosts. However, in the avian H6 subtype strains that exhibit enhanced affinity for human receptors, no mutations involving 226L and 228S were observed in HA [37]. These results indicate that there are other amino acid sites or mechanisms that play a key role in the specific binding properties of viruses.

Furthermore, research indicates that the deletion of amino acids in the stalk region of the NA protein can enhance the virulence of the influenza virus. Mutations in amino acids such as E119V and H274Y can weaken the virus's sensitivity to neuraminidase inhibitors [18]. Mutations at sites related to the activity of the PB2 protein, such as E627K, D701N, and T271A [23], can alter the virus's virulence toward the host, affecting the transcription and translation processes within host cells. The S31N mutation in the M2 protein can impact the influenza virus's resistance to adamantane-class drugs [24, 38]. The NS1 protein, serving as a determinant of virulence, plays a crucial role in the virus replication process. The NS1 protein can inhibit the host's interferon-mediated antiviral immune response. Amino acid deletions or mutations in the NS1 protein are also key factors in enhancing virulence. When the 92nd amino acid of NS1 mutates from D to E and the 42nd amino acid mutates from P to S, the virus can evade the host's interferon (IFN) and tumor necrosis factor alpha (TNF-*α*) antiviral effects [26]. This alteration also determines the virulence of porcine influenza virus toward the host, enhancing the virulence of highly pathogenic avian influenza in mice. In this study, both viruses carry the P42S mutation, and no other relevant mutations at associated sites were found. This suggests an enhanced trend in the virus's ability to resist host infections. Continuous monitoring of molecular characteristic changes in H6N6 subtype avian influenza viruses is warranted.

## 5. Conclusion

H6N6 AIVs have acquired the ability to directly infect mammals and induce noticeable symptoms. Increasing evidence suggests that under recombination conditions, these viruses can evolve into more pathogenic forms with the potential to infect humans. In addition, the findings suggest that the H6N6 AIV strains isolated in this study exhibit low pathogenicity in both mammalian and avian hosts. These findings provide valuable information for understanding the genetic diversity, potential for cross-species transmission, and pathogenicity of H6N6 AIV strains in the studied regions.

## Figures and Tables

**Table 1:** The highest nucleotide identities with the whole genome of the isolated H6N6 virus. ^*∗*^

Virus	Viral gene	Gene number	The highest homologous strain	Sequence identity (%)	Virus	Viral gene	Gene number	The highest homologous strain	Sequence identity (%)
DK/GD/W3	HA	OQ842399	A/goose/China/32/2019(H6N6)	97.35	DK/GD/L31	HA	OQ842392	A/goose/China/32/2019(H6N6)	97.65
	NA	OQ852032	A/goose/China/32/2019(H6N6)	97.18		NA	OQ852025	A/goose/China/32/2019(H6N6)	97.59
	PB1	OQ862283	A/chicken/China/31/2019(H6N2)	98.07		PB1	OQ862276	A/chicken/China/31/2019(H6N2)	98.07
	PB2	OQ861240	A/chicken/China/31/2019(H6N2)	96.14		PB2	OQ861233	A/chicken/China/31/2019(H6N2)	96.27
	PA	OQ861211	A/chicken/China/30/2019(H6N2)	97.35		PA	OQ861204	A/chicken/China/30/2019(H6N2)	96.25
	NS	OQ861191	A/chicken/China/31/2019(H6N2)	97.97		NS	OQ861184	A/chicken/China/31/2019(H6N2)	98.35
	NP	OQ861150	A/goose/China/32/2019(H6N6)	98.73		NP	OQ861143	A/chicken/China/31/2019(H6N2)	98.66
	M	OQ861130	A/goose/China/32/2019(H6N6)	98.98		M	OQ861123	A/chicken/China/31/2019(H6N2)	98.78

^*∗*^Gene accession numbers and the highest homology strains of eight genes of the virus.

**Table 2:** Key molecular marker of the isolated H6N6 virus.

Viral protein	Amino acid ^*∗*^	DK/GD/W3	DK/GD/L31	Phenotype	Reference
HA	Cleavage sites 340–348	PQIETR↓GLF	PQIETR↓GLF	Typical characteristics of low pathogenic avian influenza	[12, 13]
	A138S	A	A	Increased preference for human receptors	[14]
	E190Q	**Q**	**Q**	Determination of binding ability to mouse and human lung epithelial cells	[15, 16]
	A222V	A	A	Increased preference for human receptors	
	Q226-G228	**L**RG	**L**RG		

NA	Stalk region deletion	NO	NO	Enhancement of mammalian virulence	[17]
	E119V	E	E	Sensitive to the neuroaminidase inhibitor (Oseltamivir)	[18, 19]
	H274Y	H	H		

PB2	E627K	E	E	Enhancement of polymerase activity, thereby increasing the virulence and transmission of the virus in mammals	[20, 21, 22, 23]
	D701N	D	D		
	T271A	T	T		

M2	S31N	S	S	M2 channel blocker antiviral drugs (such as amantadine) develop resistance	[24, 25]

NS	D92E	D	D	Enhancement of virus evasion of host antiviral cellular factors	[26]
	P42S	**S**	**S**		

PA	I38M	I	I	Enhancement of virulence in mice	[27, 28]

^*∗*^H3 numbering. Bold indicates mutations at amino acid sites.

**Table 3:** Hemagglutination assays of H6N6 subtype viruses using goose red blood cells with treatment.

Virus stain	HA titers (log_2_)	
	Untreated GRBCs	Treated GRBCs
CA/04	6	6
H5/Re-5	7	—
DK/GD/W3	6	5
DK/GD/L31	5	—

“-”: No blood clotting.

**Table 4:** Replication of H6 viruses in mice.

Virus	Viral titers in organs (log_10_ EID_50_/mL)						
	Days post infection	Kidney	Spleen	Brain	Turbinate	Lung	Trachea
DK/GD/W3	3	<	<	0.33 ± 0.58	3.4 ± 0.39	4 ± 0.25	2.33 ± 1.28
	5	<	<	2.14 ± 1.88	1.89 ± 1.64	4.9 ± 4.5	3.98 ± 0.20

DK/GD/L31	3	<	<	<	2.24 ± 1.04	4 ± 0.25	1.17 ± 2.02
	5	<	<	<	1.87 ± 0.01	4.1 ± 0.1	3.81 ± 0.64

“<”: The virus was not detected in the undiluted sample; the virus titers were shown as mean ± SEM.

**Table 5:** Virus titers at 2, 4, and 6 dpi in chickens.

	Viral titers in swabs (log_10_ EID_50_/mL)					
Virus	2 dpi		4 dpi		6 dpi	
	Cloacal	Oropharyngeal	Cloacal	Oropharyngeal	Cloacal	Oropharyngeal
Mock	<	<	<	<	<	<
DK/GD/W3	1(1/3)	2.75 ± 0.25	<	<	<	<
DK/GD/L31	1.83 ± 0.56	1.47 ± 0.67	<	1.25 ± 0.33	<	<

SPF chickens were intranasally inoculated with 10^6^ EID_50_ virus, and oropharyngeal and cloacal swabs were collected on the indicated days. The virus titers were shown as mean ± SEM, Abbreviations: dpi, day postinfection. “<”: The virus was not detected in the undiluted sample.

**Table 6:** Tissue distribution of the H6N6 virus in chickens at 5 dpi.

Virus		Viral titers in organs (log_10_ EID_50_/mL)					
	Heart	Liver	Spleen	Lung	Kidney	Duodenum	Trachea
Mock	<	<	<	<	<	<	<
DK/GD/W3	<	<	<	2.17 ± 2.93	1.65 ± 1.46	<	1.8 (1/3)
DK/GD/L31	<	<	<	4.07 (1/3)	<	<	<

SPF chickens were intranasally inoculated with 10^6^ EID_50_ virus; the virus titers were shown as mean ± SEM. “<”, the virus was not detected from the undiluted sample.

**Table 7:** Virus titers at 1, 3, 5, and 7 dpi in ducks.

Virus	Viral titers in swabs (log_10_ EID_50_/mL)								
	1 dpi			3 dpi		5 dpi		7 dpi	
	Cloacal	Oropharyngeal	Cloacal		Oropharyngeal	Cloacal	Oropharyngeal	Cloacal	Oropharyngeal
Mock	<	<		<	<	<	<	<	<
DK/GD/W3	2(1/4)	2.6 ± 0.125		3.4 ± 0.72	1.43 ± 0.44	2.67 ± 0.67	<	3.13 ± 0.63	<
DK/GD/L31	3.01 ± 0.81	2.3 ± 0.58		1.75 ± 0.17	2.38 ± 0.44	3.25 ± 0.15	2.08 ± 0.36	2.63 ± 0.05	<

Ducks were intranasally inoculated with 10^6^ EID_50_ virus, and oropharyngeal and cloacal swabs were collected on the indicated days. The virus titers were shown as mean ± SEM. Abbreviations: dpi, day postinfection; “<”, the virus was not detected from the undiluted sample.

**Table 8:** Tissue distribution of H6N6 virus in ducks at 3 and 5 dpi.

Virus	Viral titers in organs (log_10_ EID_50_/mL)							
	Days post infection	Heart	Liver	Spleen	Lung	Kidney	Duodenum	Trachea
Mock	3	<	<	<	<	<	<	<
	5	<	<	<	<	<	<	<

DK/GD/W3	3	<	<	<	1.42 ± 0.38	<	1.06 ± 1	1.67 ± 0.58
	5	<	<	<	0.73 ± 0.14	<	0.67 (1/3)	1.75 ± 0.88

DK/GD/L31	3	<	<	<	1.79 ± 1.22	<	<	3.05 ± 0.39
	5	<	<	<	<	<	<	1.22 ± 1.18

Ducks were intranasally inoculated with 10^6^ EID_50_ virus; the virus titers were shown as mean ± SEM. “<”, the virus was not detected from the undiluted samp.

## Data Availability

The experimental data used to support the findings of this study are available from the corresponding author upon request.
